# Evolutionary Overview of Consumer Health Informatics: Bibliometric Study on the Web of Science from 1999 to 2019

**DOI:** 10.2196/21974

**Published:** 2021-09-09

**Authors:** Wei Ouyang, Wenzhao Xie, Zirui Xin, Haiyan He, Tingxiao Wen, Xiaoqing Peng, Pingping Dai, Yifeng Yuan, Fei Liu, Yang Chen, Aijing Luo

**Affiliations:** 1 The Third Xiangya Hospital Central South University Changsha China; 2 School of Life Sciences Central South University Changsha China; 3 Key Laboratory of Medical Information Research, Central South University, College of Hunan Province Changsha China; 4 The Second Xiangya Hospital Central South University Changsha China

**Keywords:** consumer health informatics, consumer health information, thematic evaluation, co-word analysis, informatics, SciMAT

## Abstract

**Background:**

Consumer health informatics (CHI) originated in the 1990s. With the rapid development of computer and information technology for health decision making, an increasing number of consumers have obtained health-related information through the internet, and CHI has also attracted the attention of an increasing number of scholars.

**Objective:**

The aim of this study was to analyze the research themes and evolution characteristics of different study periods and to discuss the dynamic evolution path and research theme rules in a time-series framework from the perspective of a strategy map and a data flow in CHI.

**Methods:**

The Web of Science core collection database of the Institute for Scientific Information was used as the data source to retrieve relevant articles in the field of CHI. SciMAT was used to preprocess the literature data and construct the overlapping map, evolution map, strategic diagram, and cluster network characterized by keywords. Besides, a bibliometric analysis of the general characteristics, the evolutionary characteristics of the theme, and the evolutionary path of the theme was conducted.

**Results:**

A total of 986 articles were obtained after the retrieval, and 931 articles met the document-type requirement. In the past 21 years, the number of articles increased every year, with a remarkable growth after 2015. The research content in 4 different study periods formed the following 38 themes: *patient education*, *medicine*, *needs*, and *bibliographic database* in the 1999-2003 study period; *world wide web*, *patient education*, *eHealth*, *patients*, *medication*, *terminology*, *behavior*, *technology*, and *disease* in the 2004-2008 study period; *websites*, *information seeking*, *physicians*, *attitudes*, *technology*, *risk*, *food labeling*, *patient*, *strategies*, *patient education*, and *eHealth* in the 2009-2014 study period; and *electronic medical records*, *health information seeking*, *attitudes*, *health communication*, *breast cancer*, *health literacy*, *technology*, *natural language processing*, *user-centered design*, *pharmacy*, *academic libraries*, *costs*, *internet utilization*, and *online health information* in the 2015-2019 study period. Besides, these themes formed 10 evolution paths in 3 research directions: patient education and intervention, consumer demand attitude and behavior, and internet information technology application.

**Conclusions:**

Averaging 93 publications every year since 2015, CHI research is in a rapid growth period. The research themes mainly focus on patient education, health information needs, health information search behavior, health behavior intervention, health literacy, health information technology, eHealth, and other aspects. Patient education and intervention research, consumer demand, attitude, and behavior research comprise the main theme evolution path, whose evolution process has been relatively stable. This evolution path will continue to become the research hotspot in this field. Research on the internet and information technology application is a secondary theme evolution path with development potential.

## Introduction

The concept prototype of consumer health informatics (CHI) was first proposed by Kenneth R. Thornton of the School of Health Information Science (University of Victoria) in 1994. Kenneth R. Thornton also elaborated 4 important research directions: network evolution, automation of the patient record, outcome and other quality-related databases, and consumer health education [1]. In 1995, Ferguson [2] of the Harvard University School of Medicine put forward the concept of consumer health informatics (CHI) for the first time in his paper. He pointed out that CHI is a branch of science that studies the application of computer and wireless communication technology in consumer health care [2]. Eysenbach [3] proposed that CHI is the branch of medical information that does the following: analyses the consumers’ needs for information; studies and implements methods of making information accessible to consumers; and models and integrates the consumers’ preferences into medical information systems. This definition has been cited by the academic community more than 300 times. The American Medical Informatics Association defines CHI as the field devoted to informatics from multiple consumer or patient views and which includes patient-focused informatics, health literacy, and consumer education [4]. CHI is an interdisciplinary subject that includes nursing information, public health, health promotion, health education, library science, and communication science [4].

With the rapid development of computer and information technology, the continuous popularization of the internet, and the continuous strengthening of people’s health awareness, consumption awareness, and information literacy for making health decisions, increasingly more people obtain health-related information through the internet. Because of the interdisciplinary nature of CHI, there is no consensus on the definition of CHI. However, the core content of various definitions can be summarized as follows: CHI emphasizes consumers or users as the center and takes consumers or users and computer and information technology as the research object to explore how to use computer and information technology to meet consumers’ health or medical information needs. It is a discipline that helps consumers access relevant health or medical information and make decisions about health care and health promotion.

Previous studies have analyzed and summarized the research progress of CHI. For example, Eysenbach [3] collected literature, internet information, and reports related to CHI before 2000 and summarized the research progress of CHI in the context of health care in the information age, medical knowledge delivery to consumers, the accessibility of electronic health records to patients, decision aids to support the consumers’ choices, the quality control of health information on the internet, and other aspects. Kokol et al [5] carried out a bibliometric analysis on the literature related to health informatics and electronic health for the period 1984-2015 and discussed the current research status in this field, including trends in literary production, the geographic and journal distribution, and theoretical analyses. However, their research only clusters all research topics and does not reflect the dynamic changes in research themes at different times. Zhao and Zhang [6] reviewed the literature about consumer health information seeking in social media before 2016 and discussed the characteristics of existing research from the following perspectives: the prevalence of health information seeking in social media, discussion topics emerging from health information in social media, seeking health information from online peers, social and emotional support from social media, concerns of accessing consumer health information in social media, and other aspects. However, they did not discuss the dynamic evolution path and the evolution rules of the research themes of CHI. Further, many of these studies are qualitative research. There is also considerable subjectivity in the selection of literature, identification of important topics, and prediction of research frontiers. The combination of qualitative and quantitative analysis methods can increase the objectivity, accuracy, and comprehensiveness of the research results [7]. Bibliometrics is a measurable informatic method [8], and is often used to discover top journals and authors in a field, identify research progress [9], and predict research trends [10].

Many bibliometrics visualization tools are available, such as SciMAT, CiteSpace, UCINET, HistCite, VOSviewer. The full name of SciMAT is the Science Mapping Analysis tool. In 2012, it was developed by Cobo, López-Herrera, Herrera-Viedma, and Herrera at the Department of Computer and Artificial Intelligence in Granada University, Spain. It can be used for data preprocessing, data network analysis documentation, and result visualization. It can also be used to produce 4 kinds of maps: an overlapping map, an evolution map, a strategic diagram, and a cluster network [11]. Compared with other bibliometric visualization tools, SciMAT is unique in expressing the evolution of the theme and performs excellently in longitudinal timing analysis [7].

Overall, there have been few achievements in the field of CHI research based on bibliometrics, and the dynamic evolution of the research theme of CHI has not been explored. Therefore, this article conducted a bibliometric analysis to present the evolutionary overview of CHI by using SciMAT. The main research questions of this paper are as follows:

- What was the development trend of CHI research in 1999-2019?
- What is the main research direction of CHI research?
- How does the theme of CHI research evolve?
- What are the research trends of CHI?

## Methods

### Overview

In bibliometrics, to obtain the research topic of a certain research field in a specific period, it is common to carry out keyword co-occurrence analysis and cluster analysis on the literature collected and to obtain the development path and state of the research topic through a comparative analysis of the split and fusion of research themes in different periods. This paper uses SciMAT tool to draw the research themes’ knowledge map, analyzes the research themes and evolution status of CHI in different study periods using a strategic diagram and data flow, and discusses the dynamic evolution path and evolution law of the research themes of CHI.

**Figure 1:**
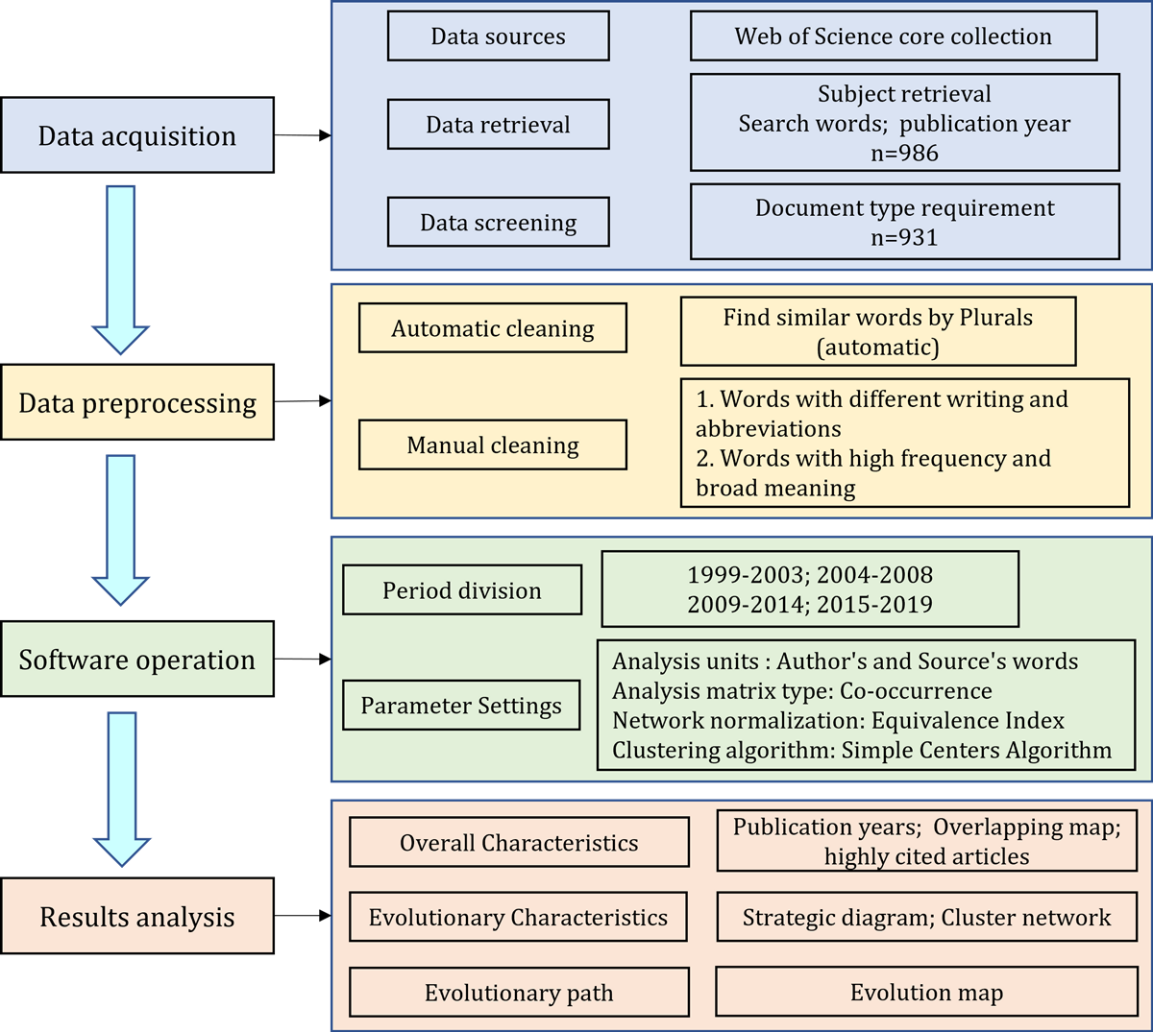
Analysis framework of consumer health informatics evolutionary.

The analysis framework of this paper is shown in Figure 1, which is mainly divided into 4 parts: data acquisition, data preprocessing, software operation, and results analysis.

### Data Sources and Retrieve Strategies

The Web of Science core collection database of the Institute for Scientific Information is an important database for obtaining global academic information [7]. It consists of the Science Citation Index Expanded, Social Sciences Citation Index, Arts & Humanities Citation Index, and so on. Users can access core academic literature from the fields of natural sciences, social sciences, biomedicine, engineering, arts, and humanities from the database. Therefore, we selected Web of Science as the data source. In this database, subject retrieval was used, and the search terms were “consumer health informatics”, “consumer health information”, “consumer medic * informatics”, “consumer medic * information”. The publication years were limited to 2019 and before. All languages were selected. Selecting all languages provides a more comprehensive coverage of the literature on this topic. Non-English articles generally have English titles, abstracts, and keywords, from which one can understand their main research content. For these articles, we also used translation software to read the full text and obtained the main research content. The literature types were limited to articles, proceeding papers, and reviews. All papers were retrieved on May 2, 2020. A total of 986 documents were obtained, of which 931 met the document-type requirement. The downloaded data were saved in a text format that SciMAT could read directly.

### Data Preprocessing

To accurately obtain the research themes in this field, it was necessary to perform data cleaning. SciMAT was used to clean keyword information. First, SciMAT’s cleaning function “find similar words by Plurals (automatic)” automatically merges the singular and plural expressions of keywords, such as “attitude“ and “attitudes” into “attitudes”. Then, similar words with different forms and abbreviations were merged by hand. For example, “consumer health information technology,” “consumer health IT,” “consumer health information technologies,” and “consumer health information technology (CHIT)” were merged into “ consumer health information technology.” synonyms, such as “physician patient relations,” “physician–patient relationship,” “patient–physician relationship,” “doctor–patient relationship,” “doctor–patient relations,” and “doctor–patient relationships” were merged into “doctor–patient relationship.” Some words, such as “consumer health information,” “consumer health informatics,” “association,” and “campaign,” which had high frequency and broad meaning and that might cover the association between other micro words, were deleted.

### Parameter Settings

Words (the author’s words and the source’s words) were selected as the units of analysis. The data reduction thresholds of the 4 study periods are 1, 1, 1, and 2, and the type of analysis matrix is co-occurrence. The network reduction thresholds are 1, 1, 1, and 1, and the network normalization method is the equivalence index. The clustering algorithm used was the simple centers algorithm. The maximum network value was 15 and the minimum network value was 3, and the scale of the cluster network was limited to a reasonable range. The H-index and sum citations were selected as the measurement indexes of clustering quality. Jaccard’s index and the Salton index were selected as similarity measurement methods for the evolution map and the overlapping map, respectively.

## Results

### Overall Characteristics Analysis

#### Trends in the Number of Articles

**Figure 2:**
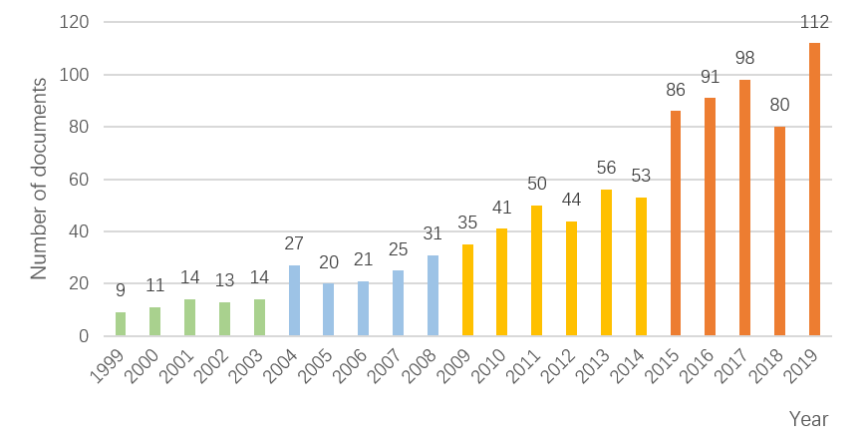
Consumer health informatic research documents published from 1999 to 2019.

The number and trend of the articles published each year in a certain field can reflect the scholars’ attention to this field. As shown in Figure 2, the amount of literature can be divided into 3 stages: slow growth, stable growth, and rapid growth. The 1999-2003 period was the first stage, with an average of 12.2 papers published each year. The period from 2004 to 2014 was the second stage, with an average of 36.6 papers published each year. The period from 2015 to 2019 was the third stage. After 2015, the number of articles increased substantially, with an average of 93.4 papers published each year. The volume of published articles peaked in 2019 at 112, with an annual total accounting for 12.0% (112/931) of all literature.

Based on the trend of literature growth, this paper divides the research on CHI into 4 study periods: 1999-2003, the first study period; 2004-2008, the second study period; 2009-2014, the third study period; and 2015-2019, the fourth study period. The number of articles in the 4 study periods is 61, 124, 279, and 467.

#### Overlapping Map Analysis

**Figure 3:**
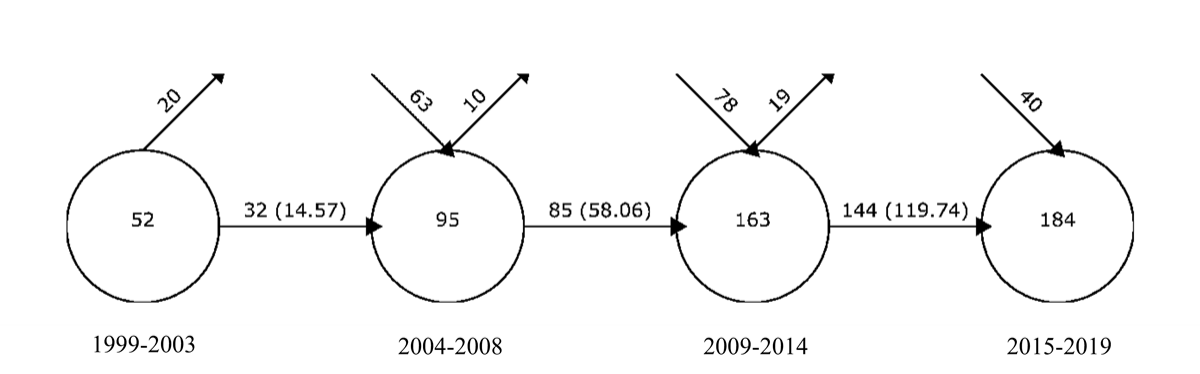
Overlapping map of the consumer health informatics research from 1999 to 2019.

An overlapping map uses the number of keywords to represent the number of themes at each period and shows the stability of a research theme in a certain field in the form of a data flow. Figure 3 is an overlapping map of CHI research for the period from 1999 to 2019 and clearly shows the emerging and declining themes in this field. The 4 circles in the figure represent the 4 study periods. Displayed from left to right, they are as follows: the 1999-2003 study period, the 2004-2008 study period, the 2009-2014 study period, and the 2015-2019 study period. The number of themes in the first, second, third, and fourth study periods is 52, 95, 163, and 184, respectively. As can be seen, the number of themes increases rapidly.

From 1999 to 2003, a total of 32 themes were passed to the next study period, and the stability index with the next study period was 14.57. In this study period, the research on CHI remains in a slow-growth stage, which is part of the basic research stage. The number of new themes in the 2004-2008 study period was 63. In the 2009-2014 period, 85 themes were continued from the previous study period, and the stability index between the 2 study periods was 58.06. Reflecting the steady growth of CHI research and the steady increase of research articles in these 2 study periods, many emerging themes and a small number of declining themes were included. There were 40 new themes in the 2015-2019 study period, and the stability index between this and the previous study period was 119.74. At this point, the number of themes is increasing steadily, which reflects that the research area of CHI is gradually expanding, and the research content is becoming increasingly richer.

#### Analysis of Highly Cited Articles

Six of the top 10 highly cited articles in this field came from the 1999-2003 study period (Table 1), and the cited frequency of the articles is ranked as the first, second, third, fifth, sixth, and tenth. This reflects that the papers published in this period form the basis of the research on CHI and play a fundamental role in the research in this field. The most frequently cited paper (940 times) is an empirical study of the quality evaluation of online consumer health information. The evaluation of health-related websites is quite different due to the differences in research methods, preciseness, quality standards, research population, and subjects. Therefore, the operability of quality standards needs to be defined [12]. The second most frequently cited (804 times) paper is the one by Cline in 2001 [13]. This paper summarizes, from the perspective of communication, the potential benefits and comprehensive quality of online consumer health information searching and identifies and discusses the criteria of online health information evaluation. In 1999, Charnock et al [14] developed a tool named DISCERN, which can be used by health information providers and consumers to judge the quality of written consumer health information about treatment options. This paper was cited 521 times. At present, DISCERN has become a highly useful tool for the quality evaluation of network health information. Many scholars use this tool to evaluate the quality of online health information about different diseases and published in different languages. For example, Cerminara et al [15] used the DISCERN tool to evaluate the reliability, accuracy, and relevance of the top 50 links for childhood epilepsy (online information) displayed by the Google search engine. Alnaim [16] used the DISCERN tool to assess the quality of information published on websites that share breast cancer information online in Arabic.

Four of the top 10 highly cited articles in the field of CHI research came from the 2004-2008 and 2009-2014 study period (Table 1). The paper “eHealth literacy: Essential Skills for Consumer Health in a Networked World” was published in the *Journal of Medical Internet Research* in 2006. In this article, the concept of eHealth literacy was defined for the first time. The ability to find, discover, understand, and evaluate health information from electronic resources, and the ability to apply the acquired knowledge to solve health problems were also explained in this paper [17]. Ranking fourth in the number of citations, the paper was cited 514 times. eHealth literacy has become a mature research topic, and increasingly more scholars are paying attention to it. For example, Kim et al [18] examined the association among eHealth literacy, perceived benefits, self-efficacy, and health-promoting behaviors in patients with type 2 diabetes. Cherid et al [19] investigated the level of mobile technology acceptance, health literacy, and electronic health literacy of 401 patients aged over 50 years with recent fractures.

**Table 1:** Top 10 cited articles in the consumer health informatics research field.

Reference	Journal	Year	Citations
Eysenbach et al [12]	*Journal of the American Medical Association*	2002	940
Cline and Haynes [13]	*Health Education Research*	2001	804
Charnock et al [14]	*Journal of Epidemiology and Community Health*	1999	521
Norman and Skinner [17]	*Journal of Medical Internet Research*	2006	514
Eysenbach [3]	*British Medical Journal*	2000	336
Gustafson et al [20]	*American Journal of Preventive Medicine*	1999	320
Or and Karsh [21]	*Journal of the American Medical Informatics Association*	2009	247
van den Berg et al [22]	*Journal of Medical Internet Research*	2007	236
Dutta-Bergman [23]	*Health Communication*	2004	229
Hibbard and Peters [24]	*Annual Review of Public Health*	2003	227

### Evolutionary Characteristics Analysis

#### Design and Overview

**Figure 4:**
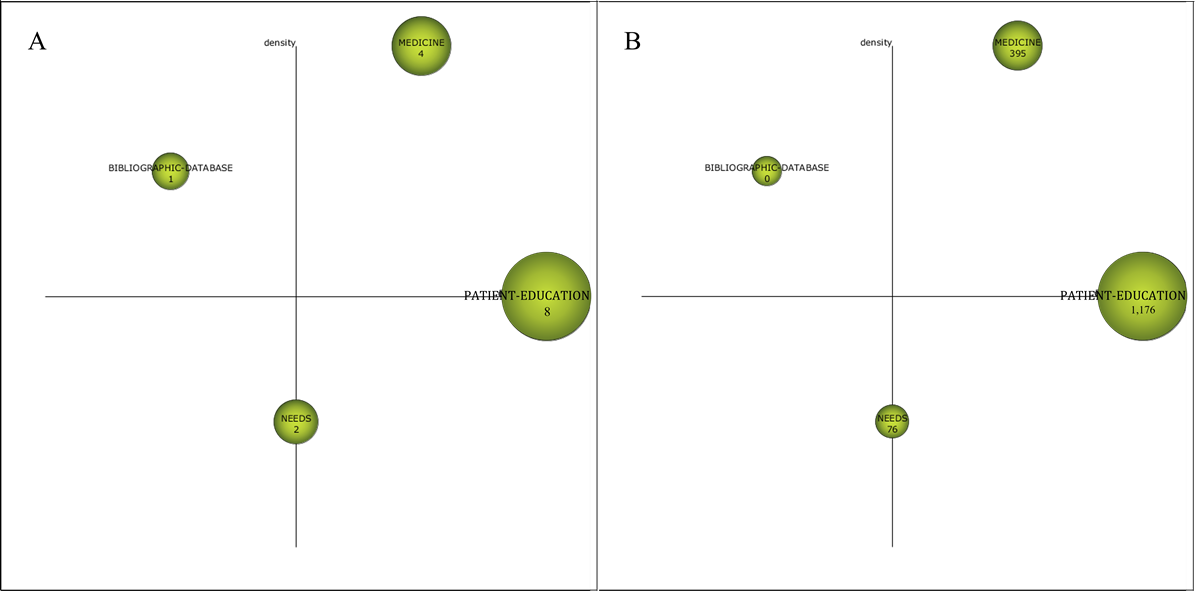
Strategic diagram of themes for the subperiod of 1999-2003 (A: based on the publications, B: based on the citation frequency).

The strategic map is mainly used to describe the relationship between themes and the relationship strength within the theme to reflect the importance of the theme in the development of the whole field and the development of the theme. The node in Figure 4 is a cluster, and the sizes of the nodes indicate the sizes of the theme clusters. The horizontal axis is the centrality, which measures the relevance of the theme to other themes. With higher centrality, the theme is more important in the whole field. The vertical axis is the density, which measures the relational strength of the cluster keywords within a theme. The higher the density of the theme, the more mature the theme. The strategic map is divided into 4 quadrants. Quadrant 1 (Q1) contains motor themes, quadrant 2 (Q2) contains highly developed and isolated themes, quadrant 3 (Q3) contains emerging or declining themes, and quadrant 4 (Q4) contains basic and transversal themes. The strategic map of the 4 study periods, the relevant bibliometrics indicators, and the evolutionary status of the research themes are as follows.

#### Themes (n=4) in 1999-2003: Patient Education, Medicine, Needs, and Bibliographic Database

**Figure 5:**
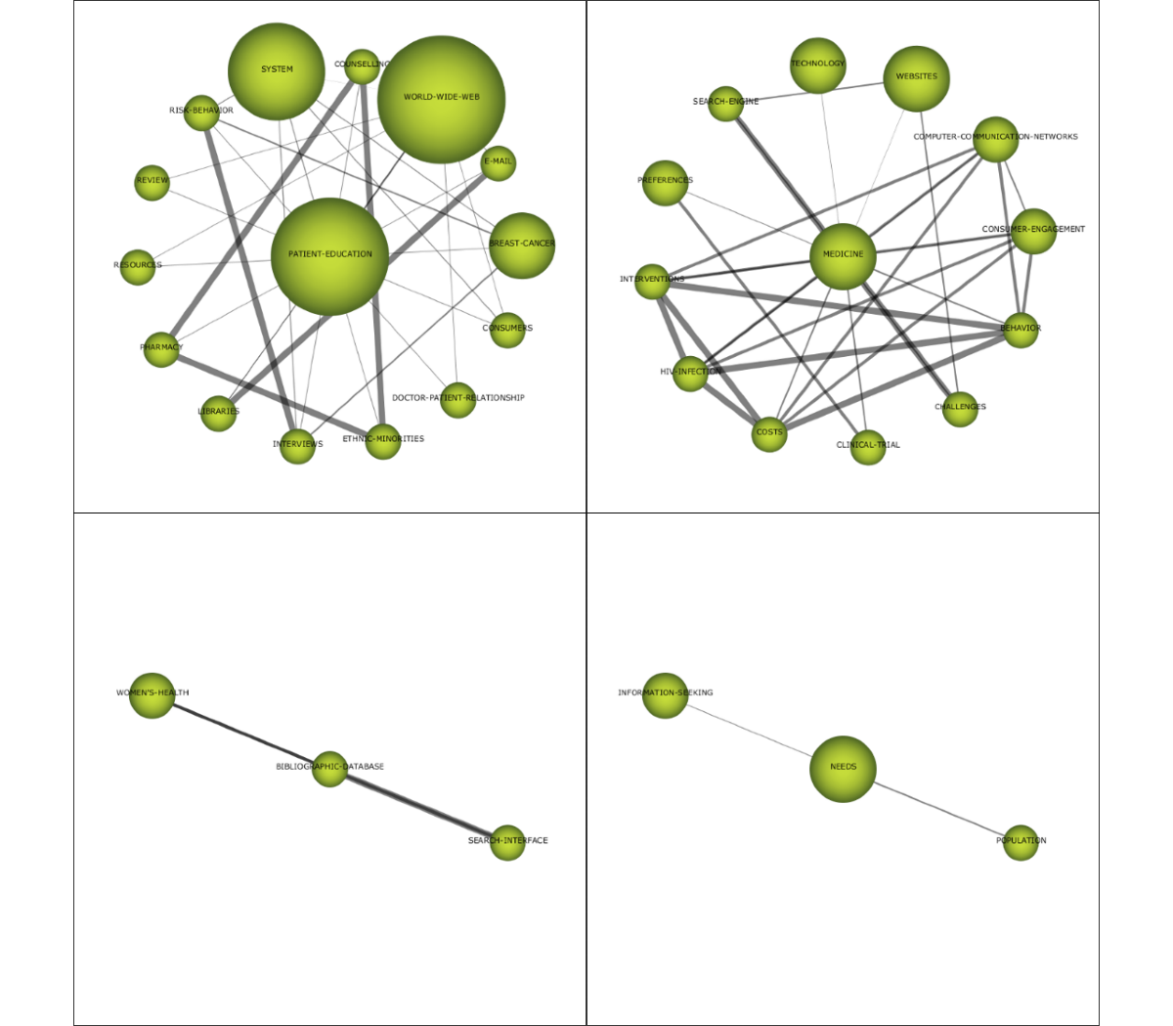
Cluster network of the themes (1999-2003).

The theme of Q1 is *medicine*, which has the highest density value and a higher centrality (Table 2 and Figure 4). The keywords closely connected to *medicine* are *search engine, interventions,* and *challenges* (Figure 5). The websites and pages displayed by search engines have become the main source from which people obtain medical and health information [25]. Health information/support systems for health promotion are also emerging, such as the CHESS comprehensive health promotion support system [20].

**Table 2:** Performance measures for the themes of the subperiod 1999-2003.

Theme	Centrality	Density	Number of documents	H-index	Number of citations
*Patient education*	45.99	56.26	8	7	1176
*Medicine*	36.24	111.7	4	3	395
*Needs*	1.85	12.5	2	2	76
*Bibliographic database*	1.25	66.67	1	0	0

The theme of Q2 is *bibliographic database*, which has centrality and density values of 1.25 and 66.67, respectively. The theme is closely related internally and has low relevance with other themes. The amount of relevant literature is the least, as the theme has not attracted the attention of the academic circle. The keywords closely connected to *bibliographic database* are *search interface* and *women’s health*. For the prototype network interface of the women’s health bibliography database, Marton et al [26] performed a comparative analysis from 3 aspects: web interface design, overall visual effect, and hypertext navigation and information organization.

*Patient education* is located on the centrality axis and has the highest centrality. *Patient education* has the largest amount of research literature and has been cited the most times, indicating that it is an area of high concern and has had a great impact on later research. *Patient education* is interconnected with many different keywords, such as world wide web and system, but the connection is not strong. The result of the cluster network shows that the internet and information systems are important tools and approaches in patient education [13] and that individualized customized education can be carried out for patients through computers [27]

*Needs* is located on the density axis and has centrality and density values of 1.85 and 12.5, respectively. The cluster network shows that *needs* is connected with *population* and *information seeking*. The internal demand for health information drives millions of consumers to search for health information on the internet. If the search terms used by consumers do not match the terms set at the information source, the search results will not meet the needs of consumers. Therefore, consumer health information retrieval needs a full range of terminology support [28].

#### Themes (n=9) in 2004-2008: World Wide Web, Patient Education, eHealth, Patients, Medication, Terminology, Behavior, Technology, and Disease

**Figure 6:**
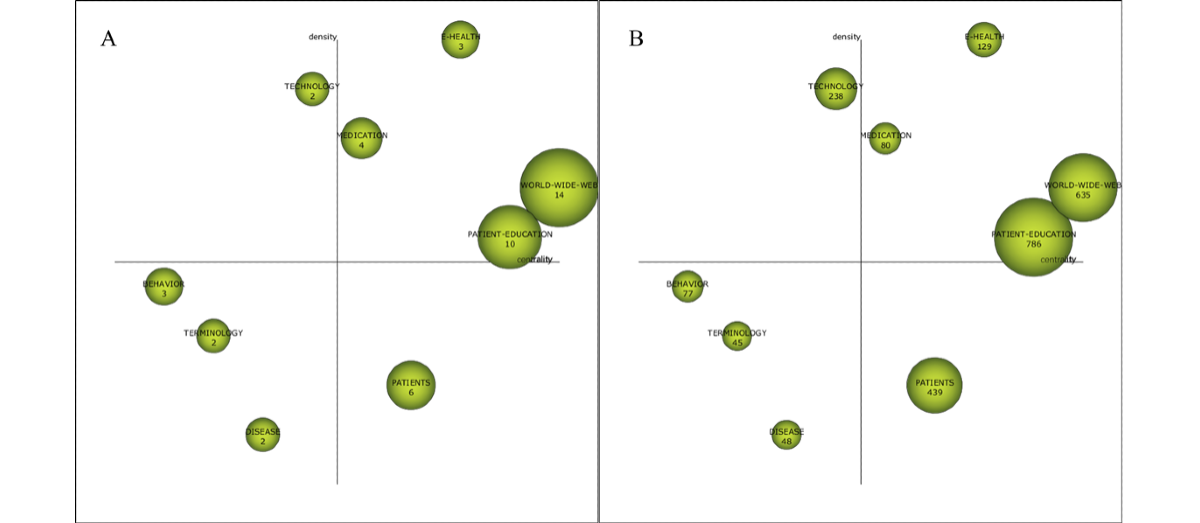
Strategic diagram of themes for the subperiod of 2004-2008 (A: based on the publications, B: based on the citation frequency).

**Figure 7:**
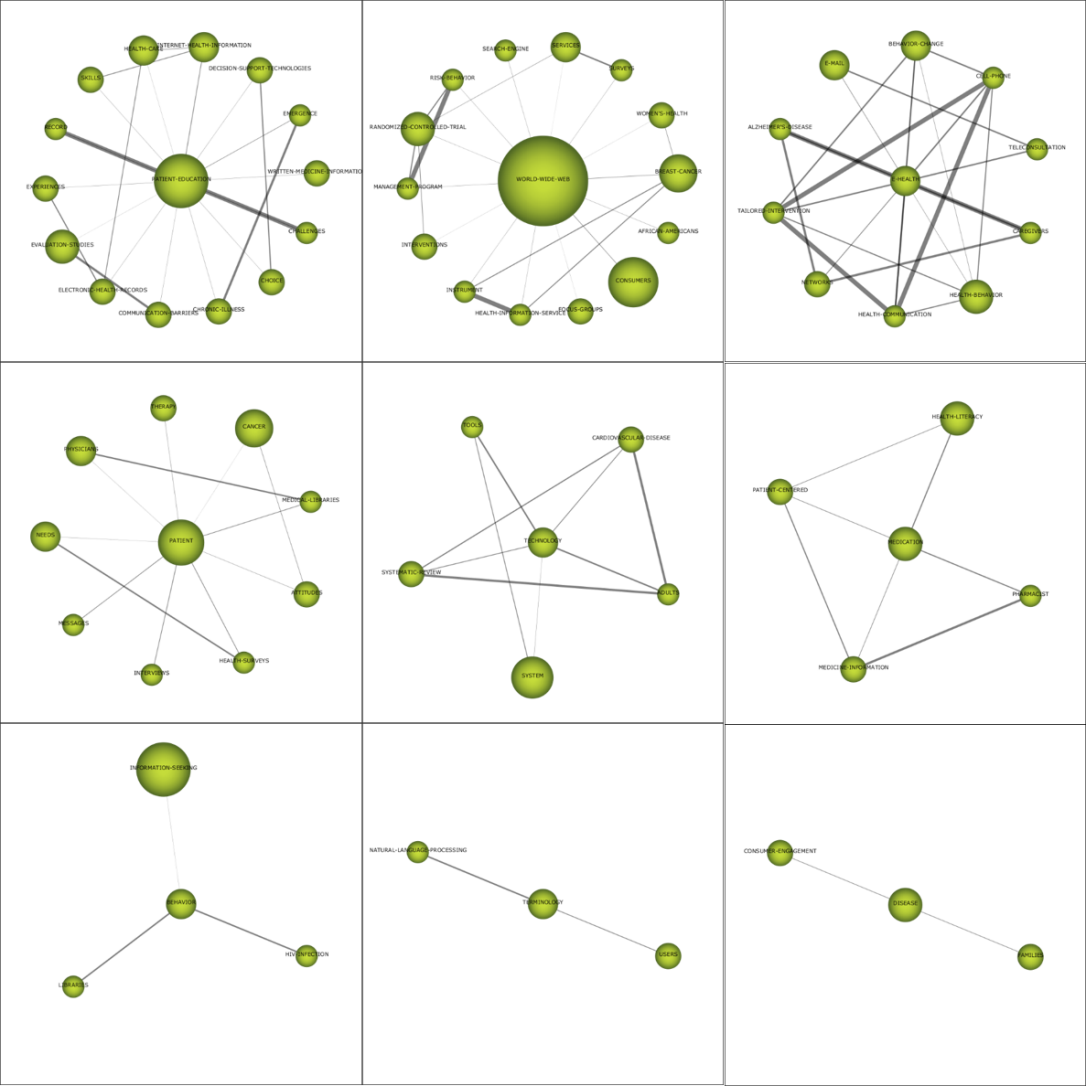
Cluster network of the themes (2004-2008).

The themes of Q1 have higher centrality and density (Table 3 and Figure 6) and are *world wide web*, *patient education*, *eHealth*, and *medication*. *World wide web* has the largest amount of research literature and has been cited 635 times. The cluster network shows that the relationship between the *world wide web* and internal keywords is weak. The keywords that are strongly connected to *patient education* are *internet health information*, *record*, and *challenges* (Figure 7). Patient education is an important part of providing health care services and helps to improve the effect of medical care. Doupi and van der Lei [29] discussed the possibility of integrating electronic medical record data and online health information resources to provide personalized patient education for patients. *eHealth* has the highest density and a relatively high centrality; the cluster network reveals that it is closely related to *health communication*, *tailored intervention, cell phone,* and *teleconsultation*, which is an important research topic in this field. Tufano and Karras [30] used teleconsultation for an obesity intervention that was expected to achieve mass customization function, interactive function, and national customized electronic health information. The location of *medication* is far from the centrality axis and close to the density axis. The cluster network shows that it has a strong relationship with *health literacy* and *pharmacist*. In the research on drug treatment in the field of CHI, scholars are concerned about the health literacy of consumers and whether they can understand the accompanying instructions of drugs [31]. Webb et al [32] have proposed that the design of patient-centered consumer medication information can improve the comprehensibility of warning labels.

**Table 3:** Performance measures for the themes of the subperiod of 2004-2008

Theme	Centrality	Density	Number of documents	H-index	Number of citations
*World wide web*	77.3	28.66	14	11	635
*Patient education*	64.37	26.23	10	8	786
*eHealth*	41.52	87.63	3	3	129
*Patients*	33.26	16.05	6	5	439
*Medication*	31.25	32.5	4	4	80
*Terminology*	3.85	16.67	2	2	45
*Behavior*	2.38	17.59	3	3	77
*Technology*	18.49	41.2	2	2	238
*Disease*	16.39	8.33	2	2	48

The theme of Q2 is *technology*, with centrality and density values of 18.49 and 41.2, respectively. The important nodes that are connected internally are *tools* and *adults*. The literature corresponding to cluster nodes reveals that scholars began using internet technology to intervene in people’s health activities [22].

The themes of Q3 are *terminology*, *behavior*, and *disease*, which have centralities of 3.85, 2.38 and 16.39, respectively, and density values of 16.67, 17.59, and 8.33, respectively. *Terminology* is only associated with *natural language processing* and *users*. The literature corresponding to *terminology* reveals that the rapid development of consumer health education websites and other applications has promoted research on consumer health vocabulary and that term *recognition* [33] is one of the important research directions. *Disease* has weak internal relations. *Behavior* is associated with *libraries*, *HIV infection*, and *information seeking*. By studying the literature corresponding to *behavior*, we found that researchers had analyzed health information searching behavior according to different populations. For example, Hesse et al [34] analyzed the 2005 Administration of the Health Information National Trends Survey data to explore the information searching behavior of survivors of cancer and found that their information searching behavior was very common and would not decrease with time.

The theme of Q4 is *patients*, with centrality and density values of 33.26 and 16.05, respectively. The internal correlation of the *patients* theme cluster was not strong. An examination of the literature corresponding to cluster nodes revealed that scholars paid more attention to the research of the patients’ attitude, cognition, and intention regarding health information [35].

#### Themes (n=11) in 2009-2014: Websites, Information Seeking, Physicians, Attitudes, Technology, Risk, Food Labeling, Patient, Strategies, Patient Education, and eHealth

**Figure 8:**
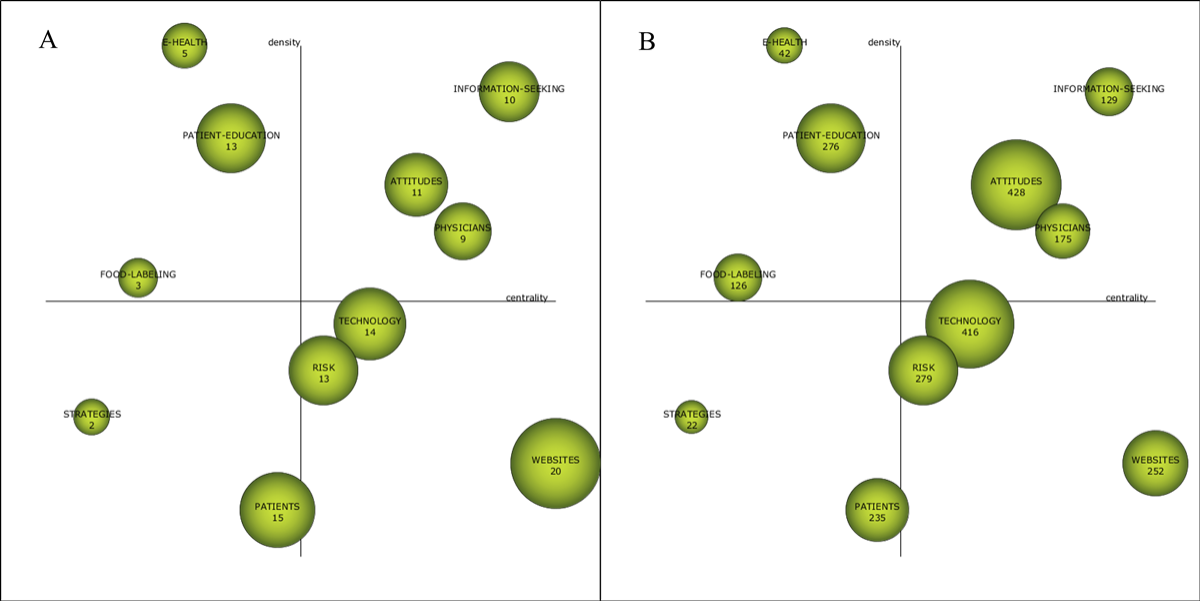
Strategic diagram of themes for the subperiod of 2009-2014 (A: based on the publications, B: based on the citation frequency).

The themes of Q1 are *information seeking*, *physicians*, and *attitudes*, which have centralities of 69.64, 67.77, and 64.83, respectively, and density values of 37.42, 19.27, and 28.02, respectively (Table 4 and Figure 8). The cluster network (Multimedia Appendix 1) shows that there are 15 nodes and 25 links in *information seeking* and that the keywords closely related to *information seeking* are *search interface*, *management program*, and *community*. The literature corresponding to *information seeking* revealed that scholars analyzed, from the perspective of consumers, the content of online questions, explored the influencing factors of health information searching, and designed a new connected exploratory navigation interface to improve the effectiveness of health information searching [36]. *Attitudes* had the highest citation frequency, indicating that it had a great influence on the subsequent research. The cluster network shows that *attitudes* is associated with a large number of different keywords, but the relationship is not strong; among these different keywords, the closely related nodes are *community pharmacies*, *information and communication techniques*, and *electronic medical records*. The cluster network reveals that the nodes that are close to *physicians* are *interviews*, *patient centered care*, *patient–provider relationship*, and *teleconsultation*. The literature corresponding to *attitudes* and *physicians* reveals that the consumers’ attitude toward, access to, and use of online information related to diseases [37], medicines, diet, and other health issues are the research hotspots in this period. For example, Amicizia et al [38] believes that information and communications technology provide an opportunity for health care workers to use mobile internet to disseminate vaccine-related knowledge interactively and entertainingly and to monitor adolescent attitudes toward vaccination through social media.

**Table 4:** Performance measures for the themes of the subperiod of 2009-2014.

Theme	Centrality	Density	Number of documents	H-index	Number of citations
*Websites*	77.12	8.45	20	9	252
*Information seeking*	69.64	37.42	10	7	129
*Physicians*	67.77	19.27	9	6	175
*Attitudes*	64.83	28.02	11	10	428
*Technology*	61.65	15.53	14	10	416
*Risk*	60.43	12.61	13	10	279
*Food labeling*	6.56	15.62	3	3	126
*Patient*	59.71	6.02	15	10	235
*Strategies*	5.5	11.11	2	2	22
*Patient education*	40.51	31.32	13	9	276
*eHealth*	30.79	37.44	5	3	42

The themes of Q2 are *patient education*, *eHealth*, and *food labeling*, which have centralities of 40.51, 30.79, and 6.56, respectively, and density values of 31.32, 37.44, and 15.62, respectively. The cluster network shows that *patient education* is closely related to *smartphone, pamphlet, resources*, and *Alzheimer’s disease*. Scholars mainly conducted research from 3 aspects: patients’ health education needs [39], the effect of health education materials, and health information technology. For example, Kraschnewski et al [40] studied how pregnant women use the internet and mobile phone technology to acquire health care knowledge. They found that women thought that the educational materials they received during prenatal care were not helpful, and therefore, they turned to the internet and smartphone apps to fill the knowledge gap [40]. There are 9 nodes and 12 connections in the *eHealth* theme. Compared with the previous study period, in this study period, there are fewer nodes and connections. The research content of the theme is more concentrated. The keywords closely related to *eHealth* are *tailored intervention*, and *online health information*. Scholars are concerned about how to use information and communication technology to help patients improve their health status and strengthen their self-health management skills [41]. For example, as a health literacy intervention for Hispanic patients with AIDS, Jacobs et al [42] developed a Spanish-language computer application, which can provide information about HIV infection, treatment, and drugs. *Food labeling* is a new theme, for which the cluster network shows the related keywords are *consumer opinions, recommendations,* and *ratings*. King et al [43] investigated the consumers’ understanding of common terms used to guide food consumption frequency and quantity and found that some terms are highly subjective and that more simple and clear terms need to be developed.

The themes of Q3 are *patients* and *strategies*, which have lower centralities and density values. The cluster network reveals that the keywords strongly related to *patients* were *health information service*, *information behavior*, and *conflict of interest*. *Strategies* is an emerging theme. The cluster network shows that the keywords associated with it are only *evidence-based practice* and *task* and that the weight of the connection is 0.17.

Having higher centralities and lower density values, the themes of Q4 are *websites*, *technology*, and *risk*. The cluster network shows that the keywords closely related to *websites* are *world wide web*, *evaluation studies*, *tools*, *email*. By studying the literature corresponding to *websites*, it is found that the research on the content evaluation of websites and health information intervention through websites are the research hotspots in this period [44]. Thakor et al [45] investigated the information quality of ecommerce websites selling *Hypericum perforatum* and found that most sites received poor reviews and lacked information on drug interactions, contraindications, and adverse reactions. The keywords closely related to *technology* are *home* *telehealth* and *aging*. With the aggravation of the aging of the population, the research on home telehealth for the aged group has attracted much attention. Cimperman et al [46] studied the important factors affecting the elderly’s adoption of home telehealth services and found that the following factors play an important role in the perception of home telehealth: perceived usefulness, expectation, social influence, perceived security, computer anxiety, convenience, and the doctor’s opinion [46,47]. *Risk* is split from *medication*. The cluster network shows that the relationship between internal keywords is weak and that the relatively strong ones are *costs*, *patient decision aid*, and *cardiovascular disease*. For veterans with and without multiple sclerosis, Cameron et al [48] studied the relative risk of falling and requiring medical care and found that the adjusted odds ratio of falling was 3 times higher for female veterans with multiple sclerosis than for female veterans without multiple sclerosis.

#### Themes (n=14) in 2015-2019: Electronic Medical Records, Health Information Seeking, Attitudes, Health Communication, Breast Cancer, Health Literacy, Technology, Natural Language Processing, User-Centered Design, Pharmacy, Academic Libraries, Costs, Internet Utilization, and Online Health Information

**Figure 9:**
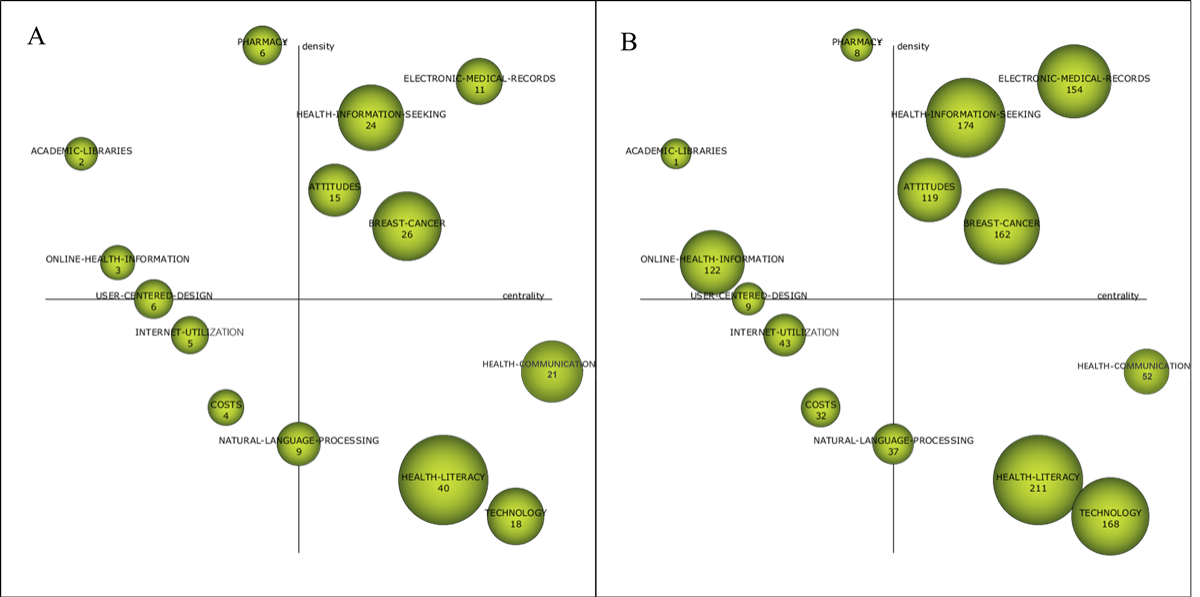
Strategic diagram of themes for the subperiod of 2015-2019 (A: based on the publications, B: based on the citation frequency).

The themes of Q1 are *electronic medical records*, *health information seeking*, *attitudes*, and *breast cancer*, with centralities of 53.52, 47.78, 45.18, and 48.08, respectively, and density values of 19.71, 18.14, 8.89, and 7.11, respectively (Table 5 and Figure 9). The cluster network (Multimedia Appendix 2) shows that *electronic medical records* has a strong relationship with internal keywords, which include *personal health information management* and *care partner*. Scholars mainly focus on the patients’ health information management, including the sharing of access to the patients’ health records, personal health data visualization, and other contents. For example, Wolff et al [49] sent the doctor’s medical records to patients and authorized nurses through OpenNotes. The scholars verified the acceptability and effect of this approach, and the results showed that it was acceptable for patients and their nursing staff to view the doctors’ medical records; the results also reflected that this method improved the communication with patients and enhanced the patients’ confidence in cooperating with nurses. Keywords closely related to *health* *information seeking* are *users*, *behavior*, *health information needs*, and *consumer health information behavior*. An examination of the literature corresponding to important nodes revealed that the users of library, online health consultation platform, social media, and other media were often considered research objects and that a discussion of these users’ health information needs and behaviors was a research hotspot in this period [50,51]. For example, taking the online health consultation case on “Taiwan eDoctor,” an online health consultation platform, as the research object, Chiu et al [52] studied the length, time, communication mode, purpose, and identity disclosure of online consultation questions and described the communication mode of patients in the process of health information searching. *Attitudes* was closely related to internal keywords, such as *HPV vaccines*, *risk*, *evaluation studies*, and *physicians*. Systematically studying the rankings, quality, and contents of the web pages related to HPV vaccines, Fu et al [53] classified the included web pages into critical and noncritical ones and found that the quality of the critical web pages was poor but that the critical web pages often obtained higher rankings. Keywords closely related to *breast cancer* are *online health* *communities*, *support groups*, and *preferences*. The scholars mainly focused on the content analysis of the online health community [54] and its supporting role for patients with cancer. For example, through interviews and questionnaires, Huh et al [55] developed online health community roles to reflect the users’ needs and requirements when using an online health community. These roles can help provide users with customized social support and patient support. The study found that the roles of online health communities can be divided into 4 categories: managers, opportunists, scientists, and adventurers. These roles reveal user interaction behavior and attitude patterns when using online health communities.

**Table 5:** Performance measures for the themes of the subperiod 2015-2019.

Theme	Centrality	Density	Number of documents	H-index	Number of citations
*Electronic medical records*	53.52	19.71	11	7	154
*Health* *information seeking*	47.78	18.14	24	5	174
*Attitudes*	45.18	8.89	15	7	119
*Health communication*	57.98	4.7	21	4	52
*Breast cancer*	48.08	7.11	26	7	162
*Health literacy*	50.29	3.55	40	8	211
*Technology*	54.79	3.46	18	7	168
*Natural language processing*	24.1	4.18	9	4	37
*User-centered design*	11.9	6.3	6	2	9
*Pharmacy*	16.52	28.49	6	2	8
*Academic libraries*	4.52	9.03	2	1	1
*Costs*	14.08	4.34	4	4	32
*Internet utilization*	12.67	4.72	5	3	43
*Online health information*	7.82	6.49	3	2	122

The themes of Q2 are *pharmacy*, *online health information*, and *academic libraries*, with centralities of 16.52, 4.52, and 7.82, respectively, and density values of 28.49, 9.03, and 6.49, respectively. A mature professional theme (*pharmacy*) has the highest density and is close to the density axis. The keyword most closely related to *pharmacy* is *patient medication information*. Monkman and Kushniruk [56] studied consumer medication information in pharmacies and found that the organization and presentation of online consumer medication information need to be improved. The content of consumer medication information needs to be improved in order to promote the safety and effective use of drugs [57]. The keywords in the *online health information* cluster network include *video*, *web* 2.0, and *world wide web*. Scholars mainly pay close attention to the use and quality evaluation of online health information [58]. As an emerging theme, *academic libraries* has the lowest centrality, the lowest amount of relevant literature, and the lowest citation frequency.

*User-centered design*, with centrality and density values of 11.9 and 6.3, respectively, is located on the centrality axis. One of the keywords clustering with *user-centered design* is *smartphone apps*. An examination of the key literature corresponding to cluster networks reveals that scholars focus on designing user-centered, smartphone-based mobile health apps, such as nutrition education apps that, for example, provide technical support for adolescents with overweight and obesity [59].

Having lower centralities and density values, the themes of Q3 are *internet utilization* and *costs*. The internal relationship between *internet utilization* and *costs* is weak; both are in an immature development stage and have little impact. The cluster network shows that the keywords in *costs* include *search engine* and *health records*; the keywords in *internet utilization* include *health behavior*, *systematic review*, and *patient education as topic*. The literature corresponding to *internet utilization* reveals that the content of relevant literature involved patients using the internet to search for health information for self-care. For example, Jamal et al [60] studied the online health information searching behavior of patients with type 2 diabetes in the Middle East and its influence on their self-care behavior. The results showed that most internet health information searchers had positive changes in their behaviors after searching and had a stronger awareness of diabetes self-care [60].

*Natural language processing* is located on the density axis, with centrality and density values of 24.1 and 4.18, respectively. The cluster network shows that *natural language processing* is connected with *consumer health vocabulary*, *state*, *online support groups*, and *text*. An examination of the core literature corresponding to the cluster shows that most scholars mined and dealt with the health-related contents of consumers through social media, health websites, health forums, and other ways to obtain the users’ health needs, explore the users’ health behaviors, and investigate the application of health information technology [61,62].

The themes of Q4 are *health communication*, *health literacy*, and *technology*, with centralities of 57.98, 50.29, and 54.79, respectively, and density values of 4.7, 3.55, and 3.46, respectively. The *technology* cluster network formed 15 nodes and 21 wires, but the internal relationship was very weak. *Health communication* has the highest centrality and has many connections with external themes. It is a key theme with development potential. The cluster network shows that *health communication* is strongly related to keywords such as *medicine*, *telehealth*, and *perspectives*. Benis et al [63] studied the use of communication channels between patients and health care organizations. By matching the communication channels with the patients’ personal information, the communication between medical institutions and patients can be transformed into a more active mode, and the patient’s participation can be improved. *Health literacy* is an emerging theme; it has the largest number of related literature and the largest citation frequency sum. The keywords closely related to *health literacy* include *skills*, *patient education*, and *public libraries*. Health literacy is the ability of individuals to access and understand health information and to use it to maintain and promote their health. An examination of the literature of important nodes reveals that scholars pay more attention to the assessment and intervention of health literacy [64] and the assessment of internet health information quality [65,66].

### Evolutionary Path Analysis

#### Design and Overview

**Figure 10:**
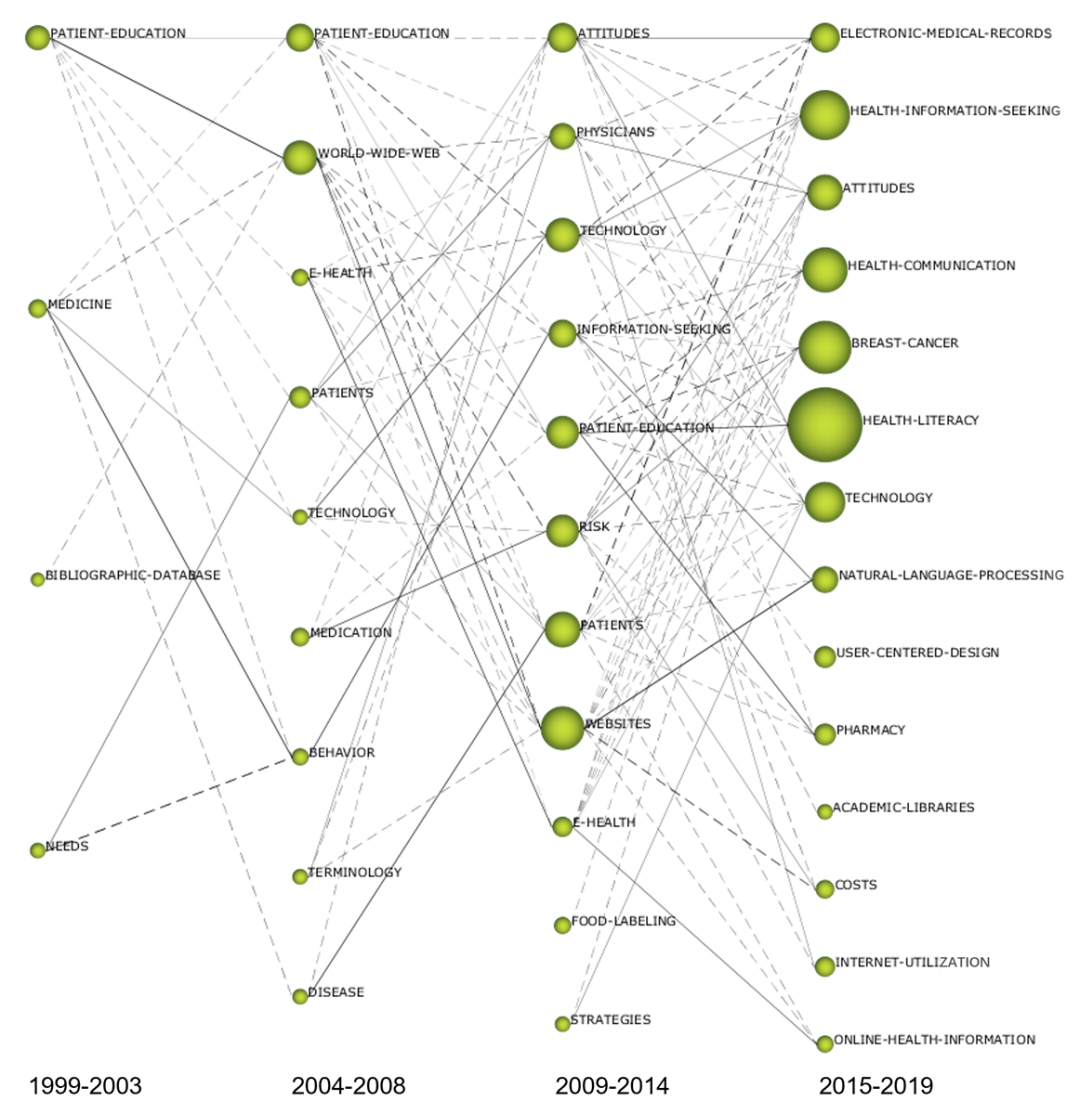
Thematic evaluation map of the consumer health informatics research field (1999-2019).

The theme evolution map shows, through a data flow, the evolution of the 4 study period themes of CHI research. It can analyze and track the dynamic evolution of the themes in the field of CHI research over a period. Figure 10 shows the CHI research field’s theme evolution map. Each column in the figure represents a study period. In the figure, the nodes represent the research themes, and the size of the nodes is proportional to the number of related articles of the research themes. The connection between the nodes represents the theme data flow, the solid line represents the main keywords shared by 2 themes, and the dotted line represents the shared keywords are not the main keywords. The width of the connection is proportional to the Inclusion index [11].

From Figure 10, it can be seen intuitively that *patient education* and *technology* appear in 3 study periods, and that *eHealth*, *attitudes*, and *patients* appear in 2 study periods. With time, the circle in the theme evolution map becomes larger, the number of the research themes increases, and the data flow between the research themes becomes increasingly complex. This reflects the emergence of new research themes and contents from 1999 to 2019. According to the theme evolution map, the size of the research theme circle, and the data flow between themes, 10 theme evolution paths of 3 research directions in the field of CHI were determined.

#### Supply-Side Research of Consumer Health Informatics: Patient Education and Intervention Research

Consumer health education is the process of assisting one to acquire the correct information and understanding so that one will be able to make wise decisions about a certain health item [67]. *Patient education* → *patient education* → *patient education* → *health literacy*, *pharmacy* (subpath 1). *Patient education* → *world wide web* → *websites* → *health communication* (subpath 2). *Patient education* → *eHealth* → *eHealth* → *health literacy*, *breast cancer*, *health communication* (subpath 3).

The 3 subpaths above evolved from the main and branch directions of the *patient education* theme in the first study period. Over time, the nodes on the evolutionary path become larger, the number of research articles increases, and the data flow from node splitting is greater. The data flow of patient education and intervention research evolution is relatively clear and represents the main path of the evolution theme of CHI. The theme on the path mainly moves between the first quadrant and the fourth quadrant, and the split and fusion of themes are obvious. In the second study period, subpaths 1 and 3 became the focus of research. In the third study period, the theme density increased and the centrality decreased, demonstrating a professional and mature research direction in the field. In the fourth study period, the themes split into several themes with great influence and development potential (*health communication* and *health literacy*). In the third study period, the subpath 2 integrated multiple themes, and the density of themes decreased. In the fourth study period, the theme split into several low-density but high centrality themes (*natural language processing*).

#### Consumer-Side Research of Consumer Health Informatics: Consumer Demand, Attitude, and Behavior

The evolutionary path of consumer demand, attitude, and behavior research consists of 3 subparts. *Needs* → *patients* → *attitudes*, *physicians* → *attitudes*, *health literacy*, *health* *information seeking*, *internet utilization* (subpath 1); *medicine*, *needs* → *behavior* → *information seeking* → *health* *information seeking*, *health literacy* (subpath 2); and *medicine* → *diseases* → *patients* → *electronic medical records* (subpath 3).

These 3 subpaths evolved from the main and branch directions of *medicine* and *needs* in the first study period. With time, the number of nodes on the evolutionary path increases, the number of research articles increases year by year, and the data flow of node splitting also increases. The themes in the evolutionary path of consumer demand, attitude, and behavior research mainly move between the first, third, and fourth quadrants, and the themes are divided and integrated. The theme of subpath 1 has gone through a stage in which centrality and density rise, and in this field, it finally forms many core themes, such as *health* *information seeking*, *health literacy*, and *attitudes*. In the second study period, subpath 2 splits to form a new theme (*behavior*). Later, this theme attracted the attention of many scholars in the field; its density and centrality increased rapidly, and it became the core theme in the field. In the second and third study periods, subpath 3 is an edge theme that is not of high concern. In the fourth study period, some themes (*attitudes, physicians, technology, patients, websites*) split and merge into a high centrality and density key theme (*electronic medical records*).

#### The Technology Research of Consumer Health Informatics: The Application Research of Internet and Information Technology

Currently, big data, internet plus, artificial intelligence, and other emerging information technologies have been tightly integrated with traditional medical and health industries. The application of internet and information technology in the field of CHI has attracted more attention from computer science and communication science scholars. The evolution path of internet and information technology application research consists of 4 subpaths. *Medicine* → *technology* → *technology* → *technology*, *health communication* (subpath 1); *patient education* → *eHealth* → *eHealth* → *online health information* (subpath 2); *needs*, *bibliographic database* → *patients*, *world wide web* → *information seeking* → *natural language processing*, *user-centered design*, *academic libraries* (subpath 3); and *medicine* → *world wide web*, *terminology* → *websites* → *online health information*, *natural language processing*, *breast cancer* (subpath 4).

These 4 subpaths evolved from the main directions of *bibliographic database* in the first study period as well as the branch directions of *patient education* and *medicine*. The number of research articles is gradually increasing, and the phenomenon of node splitting and fusion is also obvious. The theme of the evolution path of the internet and information technology application research mainly moves between the first, second, and fourth quadrants. The evolution path is relatively complex, which may be related to the application of information technology in different research directions. Subpath 1 emerged from the theme of the first study period, and the density decreased in the second study period. In the third and fourth study periods, the centrality increased. The density decreased after the split of multiple themes, and some important themes with strong influence and development potential developed. Subpath 2 divides from the theme of *patient education* in the first study period. In the second study period, a key theme with high centrality and density was formed. In the third and fourth study periods, the centrality decreased, and the density increased; it became a professional theme in the field. The data flow of subpaths 3 and 4 is relatively complex and is formed by the convergence of 2 routes in the first and second study period.

## Discussion

### Overall Development Status of Consumer Health Informatics

In the past 21 years, CHI research has been on the rise and has gone through 3 stages: slow growth, stable growth, and rapid growth. In the early stage, the development was slow, the themes were few, and the intensity was low. In the later stages, the development became diversified. After 2015, the number of research papers on CHI increased substantially, and it became a hotspot of academic research. In 2019, it reached a peak of 112 papers, a research volume accounting for 12.03% of the total literature.

The period 1999-2003 comprises the basic research stage of consumer health information. Six of the top 10 highly cited literature in this field come from this study period. This literature constitutes the cornerstone of CHI research. Consumer health information evaluation tools, such as DISCERN, that were developed in this study period have become recognized mature tools in the field. The period 2004-2014 was one of steady growth in the research on CHI. During this period, fewer keywords disappeared, and more new ones were absorbed. In 2015-2019, the number of keywords was the largest, and the research content involved was increasingly more abundant. A similarity index for the fourth study period reached a higher level, reflecting that the research field of CHI had become more mature, the inheritance of research had become stronger, and researchers were continuing to focus on more research topics.

### Evolutionary Characteristics Analysis

By analyzing the rise, decline, and the change in the density and centrality of the themes in different study periods, the evolution characteristics and status of the research themes can be reflected.

The motor themes include the following: *medicine* and *patient education* in 1999-2003; *world wide web*, *patient education*, *eHealth*, and *medication* in 2004-2008; *information seeking*, *physicians*, and *attitudes* in 2009-2014; and *electronic medical records*, *health* *information seeking*, *attitudes*, and *breast cancer* in 2015-2019. These themes have high centralities and density values. Besides, the research studies are popular and influential, and the internal relations within these themes are closely relevant. These themes are the core of the research field and their development is mature.

The highly developed and isolated themes include the following: *bibliographic database* in 1999-2003; *technology* in 2004-2008; *eHealth*, *patient education*, and *food labeling* in 2009-2014; and *pharmacy*, *academic libraries*, *online health information*, and *user-centered design* in 2015-2019. Having high-density values and low centralities, these themes do not represent the research center. They are mature and peripheral themes in this field.

The emerging or declining themes include the following: *behavior*, *terminology*, and *disease* in 2004-2008; *patient* and *strategies* in 2009-2014; and *costs* and *internet utilization* in 2015-2019. Because of their low centralities and density values, these themes have been weakly developed and are marginalized themes in this field.

The basic and transversal themes include the following: *needs* in 1999-2003; *patients* in 2004-2008; *websites*, *technology*, and *risk* in 2009-2014; and *health communication*, *breast cancer*, *health literacy*, *technology*, and *natural language processing* in 2015-2019. These themes have high centralities and low-density values, indicating that these have a high influence on the field of CHI but are not well developed.

By analyzing the evolutionary status of the themes in the 2015-2019 study period, the paper expects to predict the future development trend of the field. The centrality and density value of *electronic medical records* are extremely high. Although there are not many related documents, each document has been cited 14 times on average, indicating that it has received extremely high attention and may appear in the next research period. *Health* *information seeking* is relatively stable because of its high centrality and density value. After an internal keyword splitting and recombination, the *attitudes* theme is still high in centrality and density value, and the research enthusiasm has not decreased. It will continue to become a hot topic in the field of CHI research. *Breast cancer* split from *risk* in the previous study period, with increased centrality and density value (transferred from Q4 to Q1). With the expansion of research influence, it became more mature and eventually became the research core in the field. Although the number of studies related to *online health information* is not large, the average citation frequency of each study is the highest, indicating that it has a great influence on future research. Online health information is one of the foundations of the CHI research field, has great development potential, and may continue to appear in the next study period. *Natural language processing* has been a hot topic in recent years, and it is likely to continue to appear in the next study period. *Health literacy* is an emerging topic, with the largest number of relevant studies and total cited frequency. This indicates that it has a high level of attention and has a strong evolution and development ability. It will continue to be a hot topic in this field. *Technology* has been transferred from Q2 to Q4. Its research influence and development potential have improved, reflecting that it will continue to be the basic theme in this field.

### Evolutionary Path Analysis

By analyzing the evolution characteristics of patient education and intervention research themes, it can be found that the research in the slow growth period mainly focuses on the application and exploration of the internet and information systems and explores the health education for patients through computers and websites [13]. The research in the stable growth period mainly focuses on the use of network health information [41], the design of personalized or customized patient education [29], and the application of intelligent and mobile devices [30]. The research in the rapid growth period mainly focuses on the role and mechanism of the online health community in patient support [54], doctor–patient communication [63], and the quality evaluation of online health information [66].

By analyzing the evolution characteristics of consumer demand, attitude, and behavior research themes, it can be found that the research in the slow growth period mainly focuses on the users’ demand for online health information search, as well as demand-driven behavioral research, such as an analysis of the characteristics of health information–retrieval terms [28]. Studies in the stable growth period mainly focus on the patients’ attitude and cognition toward health information [23], health information searching behavior, and its influencing factors [68]. The research in the rapid growth period mainly focuses on personal health information management [69], health literacy assessment, and health literacy intervention [70]. The internet health information search behavior of patients with different diseases is still the research hotspot in this period.

By analyzing the evolution characteristics of the research themes regarding the internet and information technology application, it can be found that the research in the slow growth period mainly focuses on the technology applied to patient education [71] and the development of a bibliographic database network interface [26]. The studies in the stable growth period mainly focus on the use of the internet and information technology to intervene in people’s health activities [72], help improve the patients’ health status, and strengthen the patients’ self-care skills [73]. They also examined the use of information technology in the patients’ health information search behavior [74], identifying text difficulty of health information and classifying health care webpages using machine learning and natural language processing methods [75,76]. The research in the rapid growth period mainly focuses on the application of natural language processing [77] and the development and evaluation of user-centered mobile medical apps [78]. Wongchaisuwat et al [79] developed an algorithm to automatically answer health-related question by implementing a semisupervised learning algorithm. Park et al [80] analyzed the online discussion content of 3 online health communities employing text mining and k-means machine learning algorithm to compare the discussion topics.

### Limitations

In this paper, we used the SciMAT tool to analyze the keywords of the literature data and constructed the overlapping map, evolution map, strategic diagram, and a cluster network. Owing to the limitation of the scope of our school’s database, we only obtained the literature from 1999 to 2019, failed to trace the earliest research literature on CHI, and failed to build a complete evolution path of the CHI research theme.

### Conclusions

In this research, a bibliometric analysis was carried out to explore the dynamic evolution path and evolution laws of CHI research themes on a time dimension and from the perspective of strategic diagrams and data flows. CHI research focuses on themes, such as the patients’ education, health information needs, information search behavior, health behavior intervention, health literacy, health information technology, and eHealth. The research content in 4 different study periods formed the 38 themes. These themes formed 10 evolution paths in 3 research directions: patient education and intervention, consumer demand attitude and behavior, and internet information technology application. Patient education and intervention research, consumer demand, attitude, and behavior research comprise the main theme evolution path. The path’s evolution process has been relatively stable, and it will continue to represent the research hotspot in this field. Research on the internet and information technology application is a secondary theme evolution path, where obvious absorption, fragmentation, and extinction of themes have occurred. Its complex evolution process has also attracted the attention of many scholars. The research status and influence of this evolution path have gradually increased, making it a research direction with development potential.

According to the existing literature, themes that will continue to appear in the next study period include the following: *electronic medical records*, *online health information*, *health information seeking*, *attitudes*, *health literacy*, *technology*, and *natural language processing*. The first 2 themes are information resource elements *in* the consumer health information ecosystem. Because of the continuous advancement of medical informatization and the rapid development of social media, electronic health records, online health information, and online health question answering data have formed massive health information repositories. These health information big data are like gold mines waiting to be explored by researchers. The middle 3 themes are information subject elements, and the last 2 themes are the information technical elements in the environment. To maintain the sustainable development of the consumer health information ecosystem, it is necessary to start from the supply side and the demand side of health information and to solve the mismatch between service and demand by using computer and information technology.

## Appendix

Multimedia Appendix 1 Cluster network of the themes (2009-2014).

Multimedia Appendix 2 Cluster network of the themes (2015-2019).

## Abbreviations

CHI: consumer health informatics
