# Supplementary figures and images for: Evolutionary Overview of Consumer Health Informatics: Bibliometric Study on the Web of Science from 1999 to 2019
Source: J Med Internet Res. 2021 Sep 9;23(9):e21974. doi: 10.2196/21974 (PMC8461533; doi:10.2196/21974)

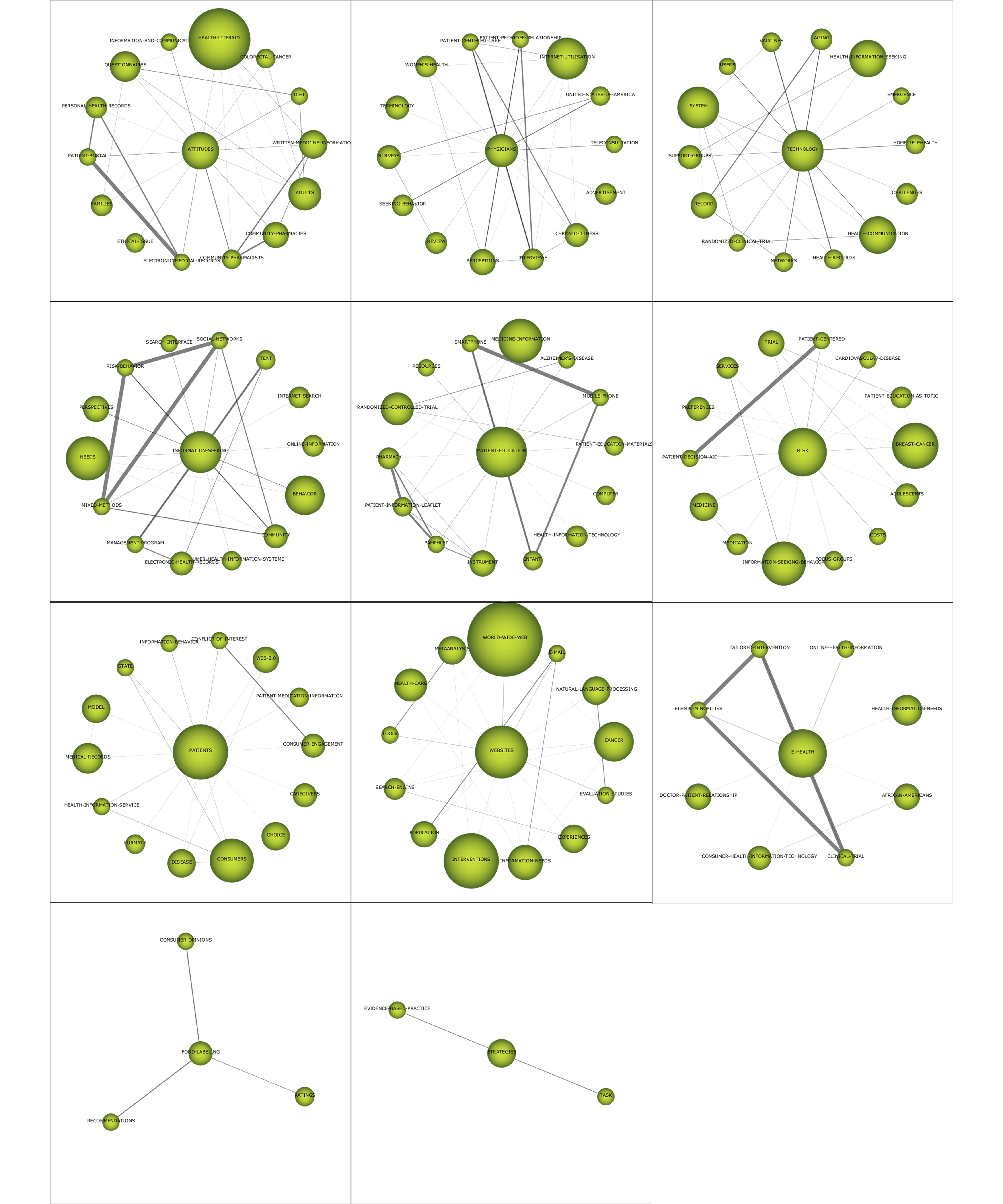

Supplement: Multimedia Appendix 1 [file jmir_v23i9e21974_app1.png]

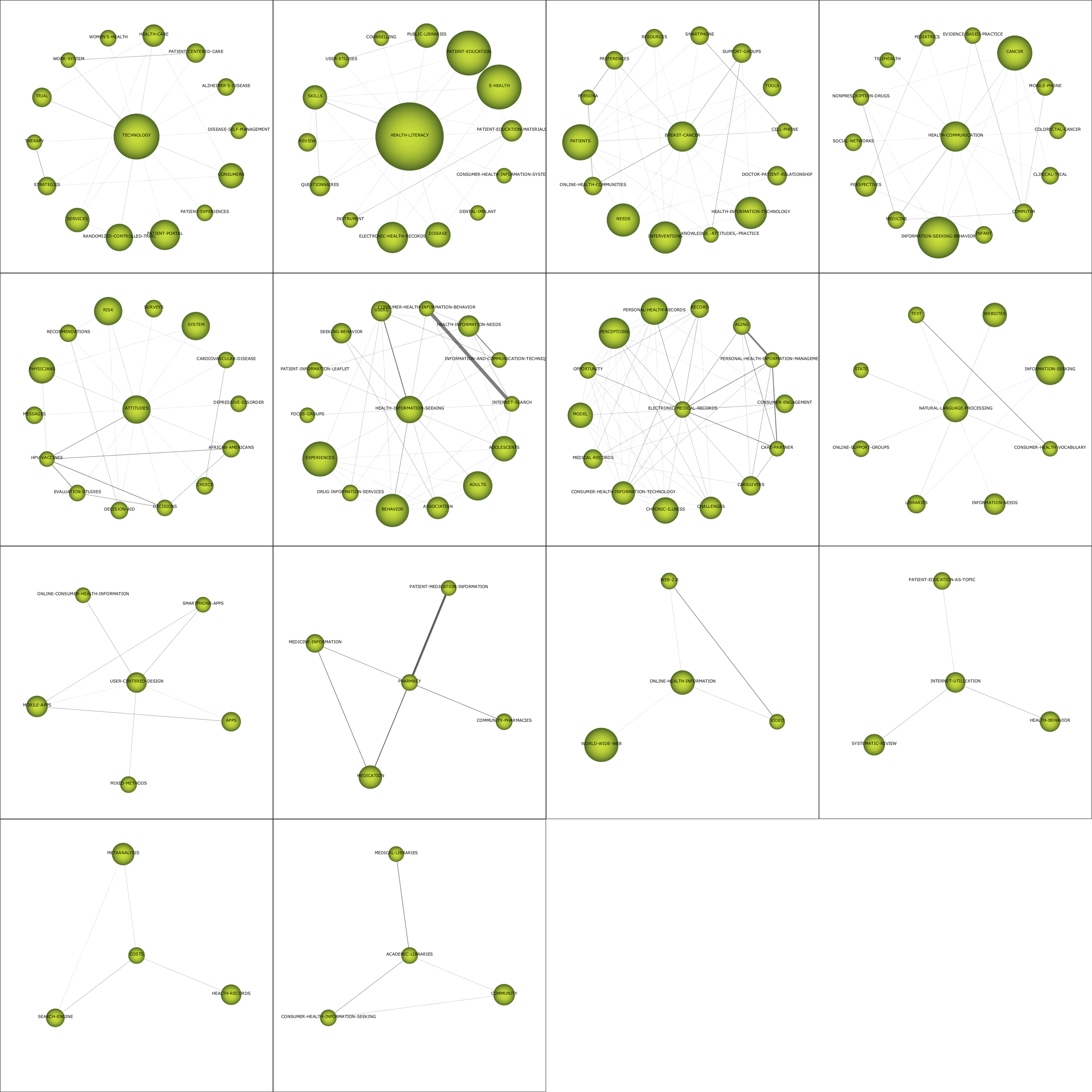

Supplement: Multimedia Appendix 2 [file jmir_v23i9e21974_app2.png]
